# Comparison of Ahmed Glaucoma Valve Implantation and Trabeculectomy for Glaucoma: A Systematic Review and Meta-Analysis

**DOI:** 10.1371/journal.pone.0118142

**Published:** 2015-02-26

**Authors:** Tan HaiBo, Kang Xin, Lu ShiHeng, Liu Lin

**Affiliations:** 1 Department of Ophthalmology, RenJi Hospital Affiliated Medical School, Shanghai Jiao tong University, Shanghai, China; 2 Department of Clinical Pharmacology, Changhai Hospital Affiliated Second Military Medical University, Shanghai, China; Casey Eye Institute, UNITED STATES

## Abstract

**Objective:**

To compare the efficacy and safety of Ahmed glaucoma valve implantation (AGV) with trabeculectomy in the management of glaucoma patients.

**Methods:**

A comprehensive literature search (PubMed, Embase, Google, and the Cochrane library) was performed, including a systematic review with meta-analysis of controlled clinical trials comparing AGV versus trabeculectomy. Efficacy estimates were the weighted mean differences (WMDs) for the percentage intraocular pressure reduction (IOPR %) from baseline to end-point, the reduction in glaucoma medications, and the odds ratios (ORs) for complete and qualified success rates. Safety estimates were the relative risks (RRs) for adverse events. All outcomes were reported with a 95% confidence interval (CI). Statistical analysis was performed using the RevMan 5.0 software.

**Results:**

Six controlled clinical trials were included in this meta-analysis. There was no significant difference between the AGV and trabeculectomy in the IOPR% (WMD = -3.04, 95% CI: -8.36- 2.26; P = 0.26). The pooled ORs comparing AGV with trabeculectomy were 0.46 (0.22, 0.99) for the complete success rate (P = 0.05) and 0.97 (0.78–1.20) for the quantified success rate (P = 0.76). No significant difference in the reduction in glaucoma medicines was observed (WMD = 0.24; 95% CI: -0.27–0.76; P = 0.35). AGV was found to be associated with a significantly lower frequency of all adverse events (RR = 0.71; 95%CI: 1.14–0.97; p = 0.001) than trabeculectomy, while the most common complications did not differ significantly (all p> 0.05).

**Conclusion:**

AGV was equivalent to trabeculectomy in reducing the IOP, the number of glaucoma medications, success rates, and rates of the most common complications. However, AGV was associated with a significantly lower frequency of overall adverse events.

## Introduction

Glaucoma is a common disease that results in blindness[1, 2]. There are various therapeutic options for treating glaucoma, including anti-glaucoma medication, laser, and surgery[3]. Trabeculectomy remains the gold standard surgical procedure for most glaucoma cases worldwide since it was first introduced[4]. Although this procedure is very effective in reducing intraocular pressure (IOP) in the short term, surgical failure has often been observed over time[5, 6]. Additionally, trabeculectomy is associated with a high incidence of early and late postoperative complications[7–10], therefore, an alternative to trabeculectomy for treating glaucoma is urgently required.

Glaucoma drainage devices were initially introduced as surgical procedures for refractory glaucoma[11,12]. Ahmed glaucoma valve implantation (AGV) is a glaucoma drainage device. The implant was equipped with a valve to reduce the occurrence of hypotony and its related complications following the early postoperative period[13]. In recent years, AGV implantation has gradually been performed as an alternative to trabeculectomy to treat all types of glaucoma.

There has been some controversy in previously published articles[13–19]on the comparative efficacy and safety of AGV versus trabeculectomy in the treatment of glaucoma. Therefore, we performed a meta-analysis of all eligible clinical trials to evaluate differences in the outcomes of the two surgical procedures for treating patients with glaucoma.

## Materials and Methods

### Search Strategy and Trial Selection

Two reviewers (Tan HB and Kang X) independently searched for related published articles using the PubMed, Embase, Google, and Cochrane Controlled Trials Register databases up to June, 2014 without restrictions on publication year or language. We used the following key terms for the search: glaucoma, glaucoma drainage devices or aqueous shunts or Ahmed glaucoma valve implant, and glaucoma surgery or glaucoma filtration surgery or trabeculectomy. Additional trials were also included after a hand search of all the references of the original reports and review articles.

Studies were considered eligible for inclusion in our meta-analysis if they met the following inclusion criteria. (1) study design: comparative clinical trials, including randomized controlled clinical trials (RCTs) and non-randomized controlled clinical trials (Non- RCTs); (2) population: patients (> four years of age) with glaucoma undergoing trabeculectomy or AGV; (3) intervention: AGV versus trabeculectomy; (4) outcome variables: at least one of the following outcome variables was included: IOPR, reduction in glaucoma medications, complete and qualified success rates, or incurrence of adverse events; and (5) duration: at least six months. The following types of studies were excluded. (1) Reviews, case reports, editorial comments, duplicate publications or letters and (2) studies that included patients with repeated or combined glaucoma surgery, other types of glaucoma surgery, and other glaucoma drainage devices.

### Data Extraction and Qualitative Assessment

Two reviewers (Tan HB and Xin K) independently extracted data. Any disagreements were resolved by discussion. The information on each eligible study included the article characteristics (authors, year of publication, and location), study design (type of study), participants (number, age, gender, race and type of glaucoma), follow up, location and baseline IOP.

The methodological quality was evaluated according to a system reported by Downs and Blacks [20]. The pilot checklist consisted of 26 items distributed between the following five sub-scales: reporting (9 items), external validity (3 items), bias (7 items), and confounding (6 items), and power (1 item). The two observers discussed any difference in the studies until a consensus was reached. The total score of each trial obtained was expressed as a percentage of the highest scores of all items counted. The trials were deemed to have adequate quality when a quality score was over 50%.

### Outcome Measures

The IOPR from baseline to end-point was calculated as described previously [21, 22]. In brief, if the mean and standard deviation (SD) of the IOPR% were reported, they were used directly. If not available, they were calculated according to the methods described below: IOPR = IOP_baseline_—IOP_end-point_ and SD_IOPR_ = (SD^2^_baseline_ + SD^2^_end-point_—SD_baseline_ * SD_end-point_) ^1/2^; then the IOPR% and SD of the IOPR% (SD_IOPR_ %) were estimated by IOPR% = IOPR / IOP_baseline,_ SD_IOPR%_ = SD_IOPR_ / IOP_baseline_.

Likewise, reduction in the number of glaucoma medications was assessed from baseline to end-point according to the methods described above. Complete success was defined as the target end-point IOP (<21 mm Hg) without medications, and qualified success was defined as the target endpoint IOP (<21 mm Hg) with or without medications. The rates of postoperative adverse events were also evaluated.

### Statistical Analysis

Analysis of the data was performed using a meta-analysis software package, RevMan (version 5.0, Information Management Systems Group, Cochrane Collaboration, and Oxford, United Kingdom). Outcome measures were assessed on an intent-to-treat basis and reported with a 95% confidence interval (CI). For dichotomous outcomes, the OR or RR was calculated. For continuous outcomes, the mean and SD were used to calculate WMD. P<0.05 was considered statistically significant for the overall effect. The heterogeneity was assessed using the I^2^ test. If there was no heterogeneity between the studies (*P* > 0.1), the random-effect model was used to assess the results in a meta-analysis. On the contrary, the random-effect model was applied for collecting the data. The standard funnel plot was constructed to inspect the published bias, and sensitive analysis was performed to confirm the stability of the meta-analysis results.

## Results

### Study Selection and Characterisitics of Eligible Studies

**Fig 1:**
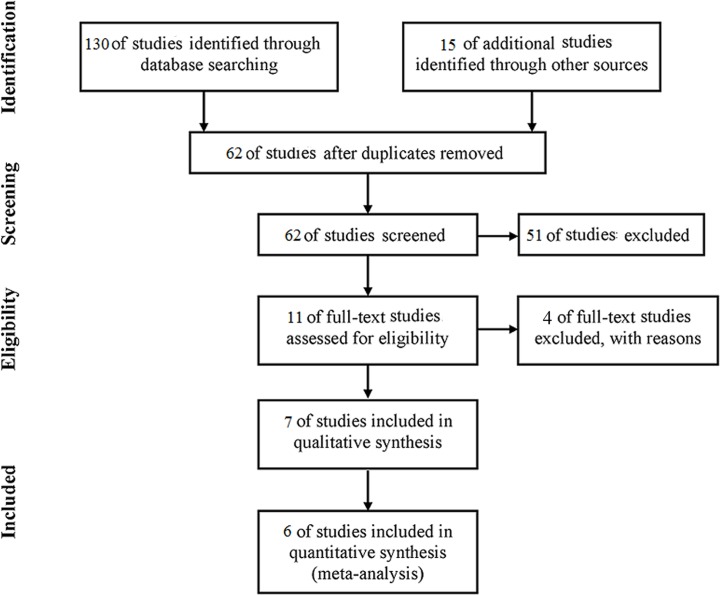
Flow chart for the selection of articles in this meta-analysis.

Fig. 1 shows the flow chart for selecting the articles. Seven relevant articles were identified by electronic and manual searches. Nine articles included in our meta-analysis were assessed. If two articles were from the same patients, the most recently published article was chosen, and one article was excluded because it employed combined trabeculectomy. Another two articles were excluded because they employed other glaucoma implant devices. Seven articles were included in the qualitative synthesis.

A total of 507 eyes from patients were included in our meta-analysis; 249 eyes were from patients in the AGV group, and 258 eyes were from patients in the trabeculectomy group. Two studies were performed in each of USA and Korean, and one was performed in each of Sri Lanka and Iran. Two of six studies had a RCT design, and four had a retrospective comparative design. The mean ages ranged from 10.9 to 69.9 years for the AGV patients and 9.1 years to 69.6 years for the trabeculectomy group patients. The Male: Female gender ratio varied from 0.44 to 4.0 in the AGV group and 0.49 to 1.0 in the trabeculectomy group. The majority of patients were Asian, followed by Caucasian, African, and Armenian patients. The mean duration of these included studies ranged from 12 to 31 months. The mean baseline IOP varied from 23.8 to 53.69 mmHg in the AGV group and 22.0 to 57.13 mmHg in the trabeculectomy group. Three of six studies reported patients with open angle glaucoma (OAG), two studies reported neovascular (NVG) cases, and there was one report each of closed angle glaucoma (CAG), aphakic glaucoma, pigmentary and pseudoexfoliative glaucoma patients. Table 1 summarizes the baseline characteristics of these included studies.

**Table 1:** Baseline Characteristics of Eligible Clinical Trials.

Author (year)	Location	Design	No.eye	Age (MD±SD)	Sex (M/F)	Type of glaucoma	Durations (mon)	Baseline IOP
Wilson(2003)	Sri Lanka	RCT	1:59 2:64	1:52.02(18.85) 2:51.92(16.39)	1:18/41 2:21/43	OAG;CAG	Mean 31	1:53.69(28.51) 2:57.13(25.02)
Im(2004)	Korean	Non-RCT	1:34 2:24	1:63.76(13.28) 2:61.47(12.41)	1:20/14 2:17/7	NVG	2–12	1:36.9(12.9) 2:39.3(12.7)
Pakravan(2007)	Iran	RCT	1:15 2:15	1:10.90(5.1) 2:9.10 (4.1)	1:12/3 2:6/7	Aphakic glaucoma	1:13.1(9.7) 2:14.8(11)	1:31(7.5) 2:31(10.7)
Lee (2008)	Korean	Non-RCT	1:27 2:41	1:56.2(12.3) 2:58.2(13.9)	1:25/2 2:32/9	Pseudophakic glaucomatous	6–12	1:34.9(8.3) 2:33.1(10.7)
Tran (2009)	USA	Non-RCT	1:94 2:94	1:69.9 (14.0) 2:69.6 (12.3)	1:42/36 2:40/48	OAG; Pigmentary glaucoma; Pseudoexfoliative glaucoma	Mean 30	1:23.8(8.5) 2:22.0 (7.2)
Shen (2011)	USA	Non-RCT	1:20 2:20	1:54. 0 (15.6) 2:59.65(15.8)	1:9/11 2:10/10	OAG; NVG	1:25.0(19.74) 2:31.05(24.45)	1:47.7(10.2) 2:47.8(11.3)

RCT: prospective randomized controlled trial; Retro: retrospective comparative controlled trial; NA: not available; AGV: Ahmed glaucoma valve implantation group; Trab: trabeculectomy group; IOP: intraocular pressure; OAG: open angle glaucoma; CAG: closed angle glaucoma; NVG: neovascular. AGV /Trab: Ahmed glaucoma valve implantation group/ trabeculectomy group.

### Quality Assessment

The qualitative assessment of these studies is summarized in Table 2. The total score for each study was above 50%. But one study[13] was not included in this meta-analysis due to a lower quality score.

**Table 2:** Quality Assessment of seven included studies.

Author (year)	Quality Score					Score	
	Reporting	External validity	Bias	Confounding	Power	Total	percentage
Wilson(2003)	11	3	5	4	3	26	81.25%
Im(2004)	9	2	3	3	3	20	62.50%
Pakravan(2007)	11	3	4	4	2	24	75.00%
Lee (2008)	10	3	3	3	3	22	68.75%
Tran (2009)	11	2	4	3	3	23	71.88%
Shen (2011)	10	2	4	3	3	22	68.75%

### Efficacy

**Fig 2:**
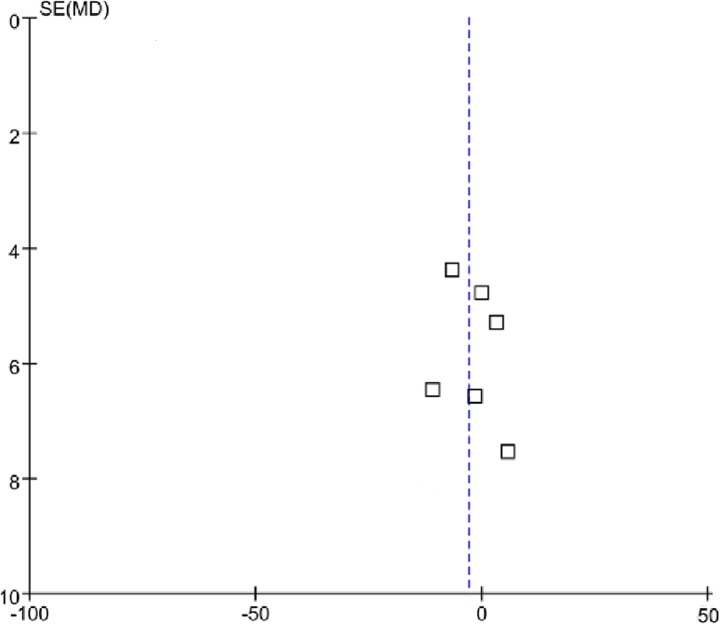
Funnel plots of studies comparing AGV with trabeculectomy in IOP reduction.

**IOP reduction** In our meta-analysis, six studies reported the mean IOP before and after surgery at various follow up time points. Because of the lack of data reported in all follow-up phases, we only compared the mean IOPR% between the AVG and trabeculectomy groups at the final follow up. The pooled result showed that both surgical procedures significantly decrease the IOP. No statistically significant difference between the AVG and trabeculectomy groups was observed in terms of the IOPR% (WMD = -3.04, 95% CI: -8.36–2.26; P = 0.26), and there was no significant heterogeneity identified in this analysis (I2 = 25%, P = 0.25) (Table 3). Subgroup analysis was performed between the two groups according to the study design (RCT and Non—RCT subgroup). The pooled results of the RCT and Non—RCT subgroups demonstrated that there was no evidence of statistically significant differences between the two groups and no significant heterogeneity was observed (Table 4). The funnel plot analysis showed no clear evidence of publication bias (Fig. 2).

**Table 3:** IOP reduction from baseline.

Studies	AGV		Trab		WMD(Fixed)(95%CI)
	No.Eyes	IOPR%:MD(SD)	No.Eyes	IOPR%(SD)	
Wilson (2003)	59	49.38(27.55)	64	49.48(25.11)	-0.10[-9.44,9.24]
Im (2004)	34	46.07(30.7)	24	58.27(30.84)	-12.20[-28.19,3.79]
Pakrvan (2007)	15	55.97(16.52)	15	52.58(1.06)	3.39[-6.96,13.74]
Lee (2008)	27	46.07(30.27)	24	56.19(29.12)	-10.92[-23.57,1.73]
Tran (2009)	94	41.18(27.55)	94	47.73(31.82)	-6.55[-15.14, 2.04]
Shen (2011)	20	71.28(24.24)	20	65.48(23.27)	5.80[-8.93,20.53]
Total	249		258		-3.04[-8.36, 2.28]
Heterogeity	I2 = 25%				
Test for overall effect	z = 1.12	p = 0.26			

IOP: intraocular pressure; IOPR%: percentage intraocular pressure reduction; CI: confidence interval; WMD: weighted mean difference; AGV: Ahmed glaucoma valve group; Trab: trabeculectomy group.

**Table 4:** Subgroup and sensitivity analysis of the effect of the study design on IOP reduction.

Study design	No.of studies	Weight	WMD (95%CI)	Heterogeity		overall effect	
				I2	P	Z	P
**RCT**	2	100.0%	1.47[-5.47,8.40]	0%	0.65	0.41	0.68
Wilson (2003)		55.1%	-1.01[-9.44,9.24				
Pakrvan (2007)		44.9%	3.39[-6.96,13.74]				
**Non-RCT**	4	100.0%	-6.22[-12.56,0.52]	17%	0.30	1.81	0.07
Im (2004)		11.6%	-12.20[-28.19,3.79]				
Lee (2008)		18.3%	-10.92[-23.57,1.73]				
Tran (2009)		38.70%	-6.55[-15.14,2.04]				
Shen (2011)		13.6%	5.80[-8.93,20.53]				

RCT: prospective randomized controlled trial; Non-RCT: retrospective non—randomized controlled clinical trials; CI: confidence interval; WMD: weighted mean difference; IOP: intraocular pressure.

Three studies reported the cumulative probability of complete success between the AVG and trabeculectomy group at the final follow up. The difference in the two groups was not statistically significant, with a pooled RR of 0.46 (0.22, 0.99) and lack of heterogeneity (I^2^ = 0%, P = 0.81) (Table 5), although the trabeculectomy group had a slightly higher number of complete successes. The results of all subgroups analysis showed no statistically difference between the two groups (p = 0.41 of RCT, p = 0.07 of Non—RCT) (Table 5). Six studies reported the cumulative probability of qualified successes between the AVG and trabeculectomy groups. There was no statistically significant difference between the two groups with a pooled RR of 0.97(0.78,1.20). No difference was observed between the two groups in the subgroup analysis based on study design [pooled RR = 1.16(0.74,1.80) of RCT, p = 0.52; RR = 0.97(0.69,1.36) of Non-RCT]. The heterogeneity test showed a lack of significant heterogeneity for the total, RCT, and Non-RCT groups (Table 5).

**Table 5:** Comparison of the Success Rate of AGV versus Trabeculectomy in the treatment of glaucoma.

No.of studies		Success Rate(n/N)		OR(95%CI)	Heterogeneity		Over Effect	
	AVG	Trab	I^2^		P	Z	P
**Completed success rate**								
Total	3	24/67	32/59	0.46[0.22,0.99]	0%	0.81	1.98	0.05
RCT	1	3/15	5/15	0.50[0.10,2.63]			0.82	0.41
Non-RCT	2	21/52	27/54	0.46[0.19,1.07]	0%	0.52	1.80	0.07
**Qualified success rate**								
Total	6	127/249	142/258	0.97[0.78,1.20]	39%	0.14	0.31	0.76
RCT	2	51/74	50/79	1.16[0.74,1.80]	43%	0.19	0.65	0.52
Non-RCT	4	82/175	94/179	0.89[0.68,1.16]	34%	0.21	0.88	0.38

RCT: prospective randomized controlled trial; Retro: retrospective; RR: Relative Risk CI: confidence interval; WMD: weighted mean difference; AGV: Ahmed glaucoma valve group; Trab: trabeculectomy group.

### Safety

Six studies reported the mean number of pro and postoperative glaucoma medications in both groups. The two groups required a similar number of postoperative glaucoma medications at the final follow up (WMD = 0.24, 95% CI: -0.27–0.76, p = 0.35). However, statistically significant heterogeneity was observed in the groups (I^2^ = 85%, P = 0.0001). Due to the larger sample size (184 eyes) found in the study by Tran et al[18], we performed sensitivity analysis after the removal of data from Tran et al[18], suggesting that the WMD of glaucoma medications reduction was 0.06 (-0.28, 0.39) and no significant heterogeneity was identified (I^2^ = 46%, P = 0.14). For RCT subgroups analysis, there were no statistically significant difference between groups (WMD = 0.09, 95% CI, -0.17–0.35, p = 0.50), and no statistical heterogeneity was found (I^2^ = 21%, P = 0.26). For the Non- RCT subgroups analysis, statistically significant heterogeneity was observed in this analysis (I^2^ = 83%, P = 0.003). The sensitivity analysis demonstrated no significant heterogeneity (I^2^ = 46%, P = 0.05). The WMD of glaucoma medication reduction was 0.10 (-0.54, 0.74) (Table 6).

**Table 6:** Reduction of Glaucoma Medications from baseline.

No.of studies	WMD(Random)(95%CI)	Heterogeity		overall effect	
		I^2^	P	Z	P
Total studies	0.24[-0.27,0.76]	85%	0.0001	0.94	0.35
Sensitivity analysis	0.06[-0.28,0.39]	46%	0.14	0.33	0.74
RCT	0.09[-0.17,0.35]	21%	0.26	0.67	0.50
Non-RCT	0.33[-0.61,1.27]	83%	0.003	0.69	0.49
Sensitivity analysis	0.10[-0.54,0.74]	76%	0.05	0.16	0.87

RCT: prospective randomized controlled trial; Non-RCT: retrospective non—randomized controlled clinical trials; RR: Relative Risk CI: confidence interval; WMD: weighted mean difference.

Six studies reported at least one postoperative complication. There were 120 complications among the 249 eyes in the AGV group and 164 complications among the 258 eyes in the trabeculectomy group. The occurrences of the most common complications in both groups are listed in Table 7. No statistically significant differences were found in the overall occurrence of all complications in the AGV group than in the trabeculectomy group [pooled RR = 0.90 (0.71, 1.14); p = 0.001], and no heterogeneity was identified (I^2^ = 0%, P = 0.34), indicating that the AGV patients experienced a lower overall occurrence of complications than the trabeculectomy patients. The incidence of bleb leakage and hypotony was numerically higher for the trabeculectomy group, on the contrast, the occurrence of corneal drying or dellen was numerically higher for the AGV group. However, the analysis of each commonly observed complication demonstrated that the difference between the two groups was not statistically significant for hypotony, hyphema, shallow or flat anterior chamber, choroidal effusion, bleb leakage, and corneal drying or dellen, and no statistically significant differences were identified (all P > 0.05) (Table 7).

**Table 7:** Risk of adverse events comparing AGV with trabeculectomy.

Adverse events	No.of studies	Crude Rate(n/N)		RR(95% CI)	Heterogeneity		Test for Over Effect
		AGV	Trab		I^2^	P	
Hypotony	2	41/141	61/155	0.87[0.30,2.49]	60%	0.08	P = 0.08
Hyphema	5	43/234	42/243	1.10[0.68,1.78]	79%	0.0007	P = 0.69
Shallow anterior chamber	4	15/135	17/144	0.98[0.43,2.23]	9%	0.35	P = 0.80
Bleb leakage	4	2/140	10/149	0.33[0.10,1.06]	0%	1.0	P = 0.06
Corneal drying/dellen	2	17/153	7/158	2.40[0.98,5.87]	5%	0.30	P = 0.06
Total events	6	120/249	164/258	0.90[0.71,1.14]	0%	0.34	P = 0.001

RCT: prospective randomized controlled trial; Retro: retrospective; RR: Relative Risk; CI: confidence interval; WMD: weighted mean difference. AGV: Ahmed glaucoma valve group; and Trab: trabeculectomy group.

## Discussion

In the present meta-analysis, six controlled clinical trials were reviewed, consisting of two RCT studies and four Non—RCT studies. After pooling the results of these trials, we found that both procedures shared similar efficacy of reduction in the IOP, number of glaucoma medications and cumulative probabilities of success. For safety, the AVG was associated with a significantly lower overall frequency of adverse events. However, both procedures resulted in similar adverse events in terms of each common complication. Sensitivity and publication bias testing indicated that the pooled results were valid.

In the present review, both procedures shared similar efficacy for reducing the IOP. The data about the IOPR% was only from a final follow up because the studies had different durations. As for the cumulative probabilities of success, we also chose the pooled data reported at the final follow up. According to the success rate criterion in our review, no significant difference was observed between the AGV and trabeculectomy groups. However, Tran et al[18] reported different results based on their criterion. Where a greater IOP reduction was required, the success rate of trabeculectomy was significantly better than AGV.

AVG influences the aqueous flow by placing a tube between the conjunctiva and sclera. Theoretically, due to the inclusion of a restrictive valve-like device, the AGV implant has the significant benefit of controlling early postoperative IOP and reducing the risk of hypotony compared to trabeculectomy[13, 23]. We found that the two groups had similar rates of complications, such as hypotony, hyphema, flat anterior chamber, and choroidal effusion. We reviewed the included articles and found that the authors did not agree on the definition of each complication. In these included studies, Wilson et al[14] did not consider an encapsulated bleb to be a complication if the IOP was less than 21 mmHg. Shen et al[19] did not consider early bleb leaks to be complications. Unlike trabeculectomy, AGV did not require iridectomy, which increased the risk of hyphema, whereas the groups did not differ in the frequency of hyphema. Additionally, there may be a risk of increased inflammation in eyes with AGV because the implants are non-human in tissues[24]. However, no anterior chamber inflammation was observed in the two groups. The varying definitions of complications may affect the results. Meanwhile, individual complications vary in severity. For example, a suprachoroidal hemorrhage can be visually devastating or self-limited without permanent sequelae. This result should be interpreted with caution because most of these complications were probably benign and self-limited. Further studies have established uniform definitions of complications and the long-term safety of the AVG in glaucoma surgery.

In clinical practice, trabeculectomy is generally preferred as the initial incisional glaucoma procedure. Glaucoma drainage implants (like the AGV) are usually used in eyes at higher risk for filtration failure. These practice patterns have been established because these two glaucoma procedures are expected to perform differently depending upon the clinical situation. Wilson’s study[14,18] evaluated the AGV and trabeculectomy in low risk eyes (i.e., POAG and PACG) without prior incisional surgery, and other studies[15,16,19] examined the two glaucoma procedures in high risk eyes (i.e., neovascular glaucoma, aphakic eyes). Therefore, the results of our meta—analysis should be interpreted with caution.

The strengths of the present meta-analysis are as follows. To minimize published bias, we identified relevant articles with computerized and manual searches, excluding articles with combined trabeculectomy, other glaucoma implant devices, a follow up of less than 6 months, and patients younger than eighteen years of age. Meanwhile, the total score for each of these included studies was over 50% according to the methodological quality assessment. Furthermore, we performed subgroup analysis based on the RCT and retrospective study design to obtain more detailed results. We also conducted sensitivity analysis and funnel plot testing to evaluate the robustness of the association results.

There are some limitations in our meta-analysis that should be taken into consideration. The first limitation was that some of these included studies were not RCTs; instead, they were retrospective non-randomized studies, which have potential sources of selection bias. Furthermore, the sample sizes of these studies were very small (range: 30 to 123), resulting in the possibility of false-negative statistical error. Additionally, the pooled data were only from the mean follow up of various studies of different durations, introducing a potential heterogeneity. Of course, publication bias was inevitable. In light of the factors described above, we must explain the results with caution. The second limitation was that the assessment criteria for the surgical success rate and complications differed between these studies. Future studies are needed to establish the standardized assessment criteria for these cases. Finally, because of the limited number of studies available in the analysis, we did not perform subgroup analysis in terms of the types of glaucoma, risks of surgical failure, race, age, and use of MMC.

Despite the limitations, our results showed that both the AVG and trabeculectomy procedures had similar efficacy of reduction in the IOP and number of medications. The AVG was associated with similar surgical success rates with a lower overall incidence of adverse events compared to trabeculectomy. However, there is still an urgent need for pragmatic RCT with long duration and a large sample size to further determine the efficacy and safety of AVG in the treatment of glaucoma.

## Supporting Information

S1 Checklist Checklist of items to include when reporting a systematic review or meta-analysis. (DOC)
